# DUNEScan: a web server for uncertainty estimation in skin cancer detection with deep neural networks

**DOI:** 10.1038/s41598-021-03889-2

**Published:** 2022-01-07

**Authors:** Bogdan Mazoure, Alexander Mazoure, Jocelyn Bédard, Vladimir Makarenkov

**Affiliations:** 1 School of Computer Science, McGill University and Quebec AI Institute (MILA), Montreal, Canada; 2 Département d’Informatique, Université du Québec à Montréal, Montreal, Canada; 3 JACOBB Applied Artificial Intelligence Center, Montreal, Canada

**Keywords:** Skin cancer, Computational biology and bioinformatics, Image processing, Machine learning, Software

## Abstract

Recent years have seen a steep rise in the number of skin cancer detection applications. While modern advances in deep learning made possible reaching new heights in terms of classification accuracy, no publicly available skin cancer detection software provide confidence estimates for these predictions. We present *DUNEScan* (Deep Uncertainty Estimation for Skin Cancer), a web server that performs an intuitive in-depth analysis of uncertainty in commonly used skin cancer classification models based on convolutional neural networks (CNNs). *DUNEScan* allows users to upload a skin lesion image, and quickly compares the mean and the variance estimates provided by a number of new and traditional CNN models. Moreover, our web server uses the Grad-CAM and UMAP algorithms to visualize the classification manifold for the user’s input, hence providing crucial information about its closeness to skin lesion images  from the popular ISIC database. *DUNEScan* is freely available at: https://www.dunescan.org.

## Introduction

Skin cancer is among the most dangerous and frequent diseases around the world. For example, in the United States alone, up to 9500 people are being diagnosed with it daily^1^. Naturally, the demand for an accurate diagnosis of skin cancer has risen through the past years  as dermatologists are facing an increasingly high number of diagnostic challenges. As a result, several skin cancer detection applications have been developed over the past few years^2–4^. Many of them leverage recent breakthroughs in deep learning architectures to achieve cutting-edge performance, often surpassing expert-level diagnosis accuracy not only in skin cancer^5–8^, but also in other pathologies^9,10^. For instance, convolutional neural networks have been able to match dermatologist-level classification accuracy only after the appearance of important computer vision breakthroughs, such as residual connections^11^ and the availability of large amounts of labeled skin lesion data. However, an increasing number of studies suggest that many popular skin cancer detection applications feature proprietary models, making it hard to assess their true performance on external datasets. Moreover, to the best of our knowledge, no publicly available skin cancer detection applications provide confidence estimates for predicted outcomes. Furthermore, models such as those used in^11^ have been trained on skin lesion images originating from multiple datasets, a practice known to increase the prediction variance due to non-standardized data pre-processing^12^. Here, we present a novel web server, called *DUNEScan* (Deep Uncertainty Estimation for Skin Cancer), which addresses the aforementioned lack of uncertainty estimates in skin cancer classification models. *DUNEScan* can be used by the domain experts (i.e. dermatologists and health practitioners), who can combine the confidence estimates of the classifier with their own observations to ensure a more grounded diagnosis. The confidence interval estimates of the classifiers provided by DUNEScan reflect the approximate Bayesian posterior of the skin lesion being either malignant or benign. If the confidence interval for a certain classifier is large, then the prediction of this classifier cannot be trusted, and hence the domain expert should rely on traditional diagnostic methods. If the confidence interval is small, then the prediction of the classifier should be taken into account and compared in detail with the diagnosis obtained using traditional methods (see the practical examples in the Results section). Our new server can be used to assess the confidence level of state-of-the-art skin cancer detection models and to visualize the related results.

*DUNEScan* features six state-of-the-art convolutional neural network models, which are used to form a consensus prediction for a given skin lesion image. What distinguishes it from existing skin cancer classification tools is that *DUNEScan* approximates the confidence (or uncertainty) of each model’s prediction for a given skin lesion image. As we show further in the paper, high-probability predictions do not necessarily imply high confidence, and therefore the model’s average prediction cannot be directly used to get an accurate diagnosis without first examining the approximate predictive posterior.

The main contributions of our work are as follows:

- We present a novel web server for accurate image-based skin cancer detection;
- Our server features six state-of-the-art convolutional neural network (CNN) models, which have been successfully used for medical predictions in the past;
- We trained our models on a complete set of skin lesion images from the popular ISIC database;
- We provide multiple ways to analyze uncertainty of CNN models predictions, including GradCAM, UMAP and Binary Dropout techniques;
- In contrast to existing software, our web server allows one to compare the average model prediction with the approximate posterior obtained with binary dropout—this comparison is critical for providing precise skin cancer diagnoses.

## Results

Deep learning-based computer vision has recently experienced immense breakthroughs. This has had a great impact on all related application domains including medical imaging. Keeping up with the latest state-of-the-art algorithms can often be challenging and time-consuming, which is why *DUNEScan* includes the most recent and best performing supervised and self-supervised methods. Moreover, since the user’s privacy and data security are especially important in digital healthcare, all web connections are performed over secured protocols.

### Available deep learning models

Our web server features six efficient CNN models, including the winners of the dermatological Kaggle competition, which are based on MobileNetv2 and EfficientNet (2019–2020). They are as follows: Inceptionv3^13^, ResNet50^14^, MobileNetv2^3^, EfficientNet^15^, BYOL^16^ and SwAV^17^. The model repository features both supervised and self-supervised models.

Inceptionv3 features a combination of small asymmetric convolutions, which results in computationally efficient operations in terms of the number of parameters, as well as memory footprint. ResNet50 uses a mechanism known as residual connections to mitigate the issue of vanishing gradients, therefore allowing one to train increasingly deeper models. Thanks to those residual connections, the ResNet50 model has become very popular in most computer vision domains, e.g. medical imaging, text-to-image translation and pixel-based reinforcement learning. MobileNetv2 greatly reduces the size and the inference time of Inceptionv3 by replacing the standard convolutional layer by depth wise convolutions, which operate on a single channel at a time. The small parameter count of MobileNetv2 allows for fast image classification, which makes it the model of choice for web-based applications and mobile devices. The EfficientNet family of models (B0 through B7) includes a set of network architectures which can be progressively combined to obtain models of higher performance, at the cost of computational complexity. Bootstrap Your Own Latent (BYOL) relies on fully unsupervised training of a ResNet network by predicting exponentially weighted averages of data augmented copies of a given image directly from the latent representation, nearly matching the performance of a fully supervised ResNet model. Swapping Assignments between multiple Views (SwAV) relies on clustering to first map the input image into one of the possible image prototypes, and then enforces predictivity of labels from clusters. Both BYOL and SwAV models are pre-trained with large amounts of unlabeled data, which makes them an excellent choice in healthcare areas, where annotating images is a costly process.

Although recent self-supervised learning models can match the performance of supervised learning models, no skin cancer detection applications have integrated self-supervised models in their pipelines so far. The major advantage of self-supervised methods is the ability to leverage large amounts of unlabeled data to pretrain the latent representation, which can then be used to train a simple classifier, matching the accuracy of fully supervised methods^16^.

### Comparative analysis of the models

The performance of convolutional models aiming at skin cancer detection depends on multiple important factors such as structure of the dataset, structure of the model, and training procedure^11^. In particular, the nature of the training dataset implicitly advantages certain types of CNN architectures over others (e.g., overwhelming presence of artifacts such as body hairs in skin lesions advantages models with residual connections, which process such fine-grained features better than the Inception-like models) as pointed out in^18^. For instance, in their study^18^, found that the ResNet models had a higher specificity than the Inception-based architectures. Lightweight CNN models, such as MobileNetv2^3^ and EfficientNet-B0^15^, prioritize fast inference time and low memory complexity over performance by simplifying large architectures. Moreover, the depth and width of a CNN model, as well as the presence of residual connections has been shown to affect the approximate predictive posterior distribution, for uncertainty estimation methods such as MC dropout, input bootstrapping, and Gaussian mixture models^19^. The uncertainty of the approximate Bayesian posterior can be estimated using the dropout technique for any deep neural networks. DUNEScan features six state-of-the-art CNN models used for supervised and self-supervised image classification. However, even these models should be used with care since their average prediction cannot be trusted in all cases.

### Comparison with traditional ML models

In this work, we discuss how *DUNEScan* provides access to large CNN models, which themselves belong to the wider family of deep learning models, trained via stochastic gradient descent. However, traditional machine learning models for classification such as decision trees or support vector machines^20^ can also accomplish the task. Multiple previous studies have provided thorough performance comparisons between CNN and traditional ML models (e.g., see^21^), and highlighted that, under extensive hyperparameter tuning, both families of methods achieve similar performance. However, the CNN models do not require handcrafted features, and can be trained in a distributed manner, which makes them more suitable candidates in the absence of expert-level data annotations.

### Uncertainty estimation

In risk-sensitive fields such as medical imaging, where a false negative prediction can make a difference between life and death, it is crucial to quantify the confidence level of a given model. *DUNEScan* uses the technique proposed by^22^, randomly disabling parameters of the classifier in an independent set of replicates, and thus achieving an approximate Bayesian posterior over the possible estimates of the model for a given skin lesion image.

**Figure 1:**
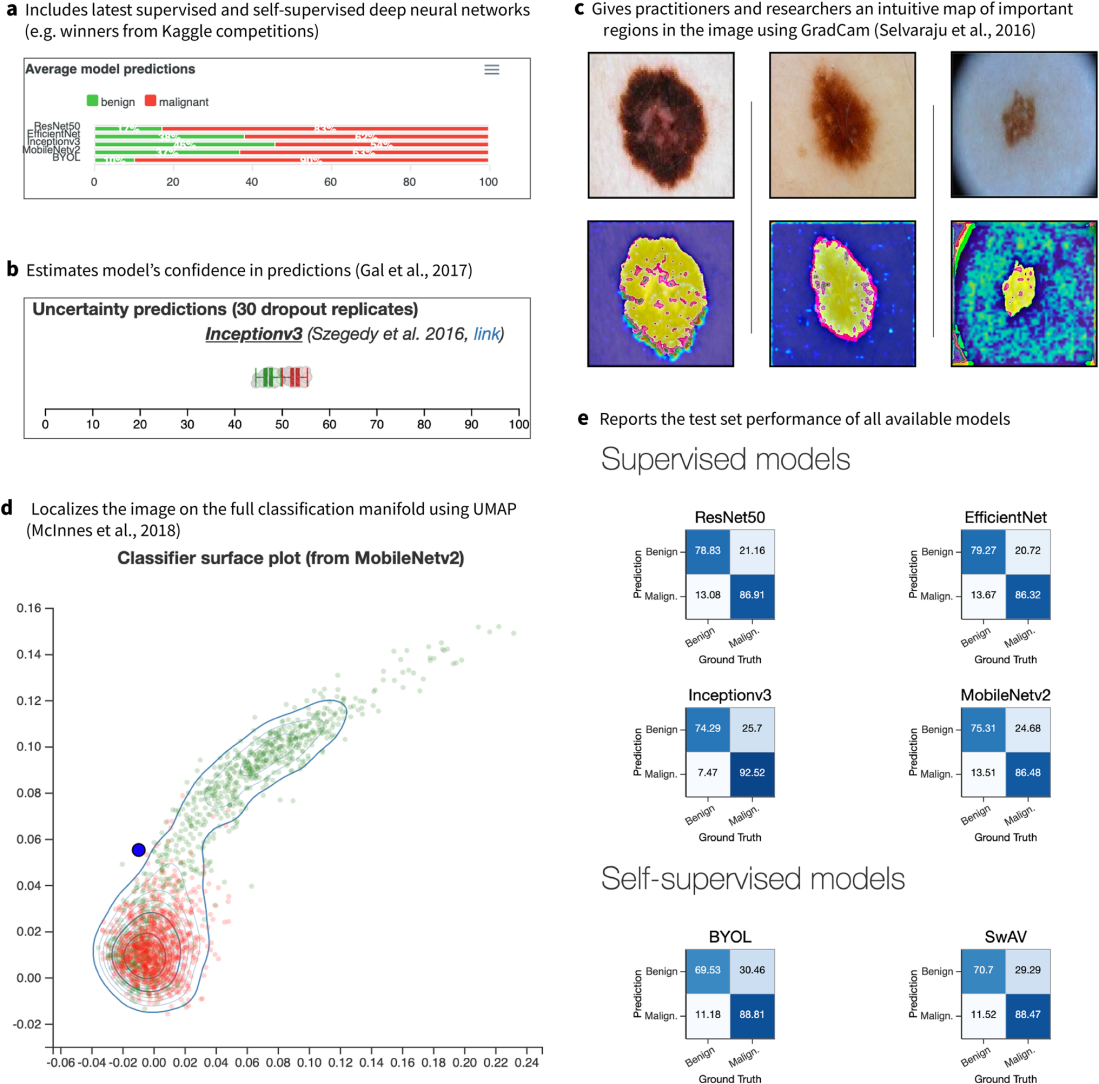
Screenshots of the main features of our *DUNEScan* web server. (**a**) Average model predictions for a given skin lesion image (malignant or benign) provided by the six available CNN models, (**b**) boxplots showing uncertainty of model predictions, (**c**) Grad-CAM gradient saliency plot of most important lesion features, (**d**) classification manifold from the MobileNetv2 features, (**e**) confusion matrices computed over the test set for all six CNN models.

The *DUNEScan* user can select the number of random replicates to be used for a given model. *DUNEScan* provides uncertainty estimates for each classifier through a boxplot (see Fig. 1b). If the prediction probabilities with the replicates are tightly concentrated around the mean, this implies that the classifier is confident in its class prediction for the input image and the prediction is trustworthy. In contrast, if the prediction probabilities for the benign and malignant image classes are dispersed and their confidence intervals overlap, this implies that the classifier is not confident and hence, the prediction is not trustworthy.

In addition to the boxplots described above, a classification manifold is also produced with the trained MobileNetv2 model, the fastest of the six available models (see Fig. 1d). This plot provides an alternative illustration of the confidence of the MobileNetv2 classifier obtained for the input image class prediction.

In the classification manifold graph, each green dot represents a benign skin lesion image used for training, and each red dot represents a malignant one (see Fig. 1d). If the input image, represented by a blue dot, is located close to the middle of the benign (green) cluster—then the MobileNetv2 model is confident that the lesion is benign, but if it is located close to the middle of the malignant (red) cluster—then the MobileNetv2 model is confident that the lesion is malignant. However, if the blue dot is located close to the boundary of the green and red clusters, then the model exhibits uncertainty in the prediction.

### Description of DUNEScan’s output

*DUNEScan* first produces and presents the output plot of Grad-CAM^23^ that highlights the regions of high importance on the input image detected by the MobileNetv2 model (see Fig. 1c). The above described MobileNetv2 classification manifold is then presented, followed by the uncertainty estimate boxplot for each model selected to analyze the input image (see Fig. 1b).

Moreover, the output contains a bar-graph showing the average prediction probabilities of both classes obtained with each model used (see Fig. 1a). By providing the classification probabilities together with means to assess the confidence of these predictions, the DUNEScan server allows practitioners to quickly evaluate the probability that a given skin lesion is benign or malignant. This probability is computed by passing a given skin lesion image through one of the six available models, which outputs a vector of 2 real values (i.e. logits). These values are passed through the softmax function, which maps them onto the probability simplex. Hence, all probabilities computed in the paper are of the form: $P[malignant|skin\;lesion\;image]$.

### Testing the application

Our application was tested by using images from the HAM10000 dataset^24^. This dataset was used as source data for the International Skin Imaging Collaboration (ISIC) 2018 challenge^25^ and includes images of skin lesions corresponding to seven different classes: actinic keratosis (akiec), basal cell carcinoma (bcc), benign keratosis (bkl), dermatofibroma (df), melanocytic nevi (nv), melanoma (mel) and vascular lesions (vasc).

Amongst these, melanoma and basal cell carcinoma are considered to be malignant skin diseases, whereas the other lesion types are considered as benign. The class labels assigned for more than 50% of the images were confirmed by histopathology, while for the others the labels were derived from expert consensus or confirmed by in-vivo confocal microscopy. Selected images were analyzed using 50 replicates with all six CNN models available in *DUNEScan* to give an overall classification prediction.

Melanoma and melanocytic nevi images, the most common malignant and benign classes of lesions in the dataset, representing ~ 11% and ~ 67% of the dataset, respectively, were used to assess the performance of the application. In general, the prediction average and the confidence in the prediction vary between the different algorithms. However, in most cases they broadly tend to agree on the prediction with some exceptions.

**Figure 2:**
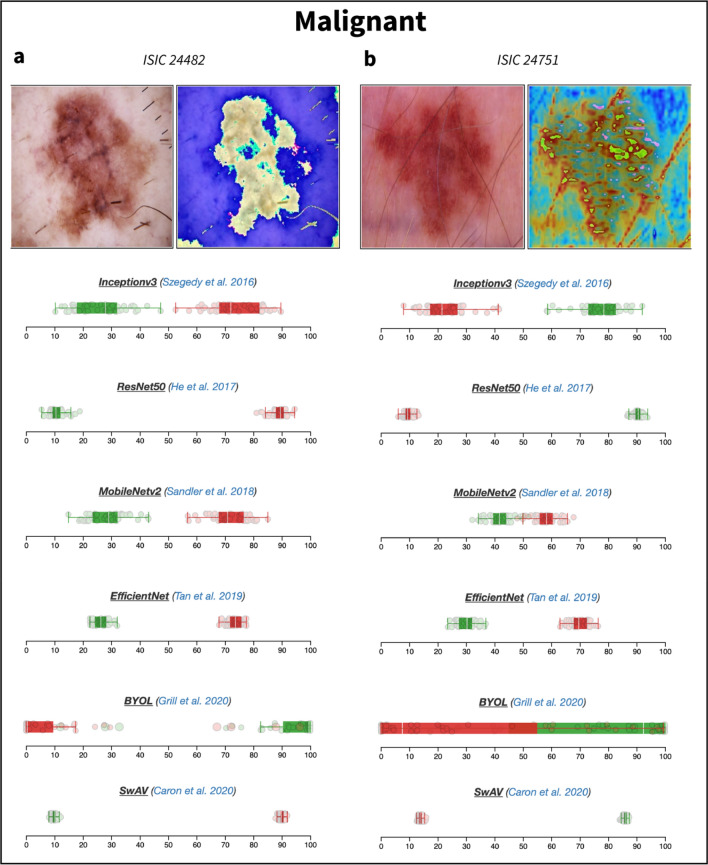
Boxplots representing uncertainty estimates provided by the six CNN models available on DUNEScan for the following skin lesion images: ISIC_0024482 (**a**) and ISIC_0024751 (**b**).

**Figure 3:**
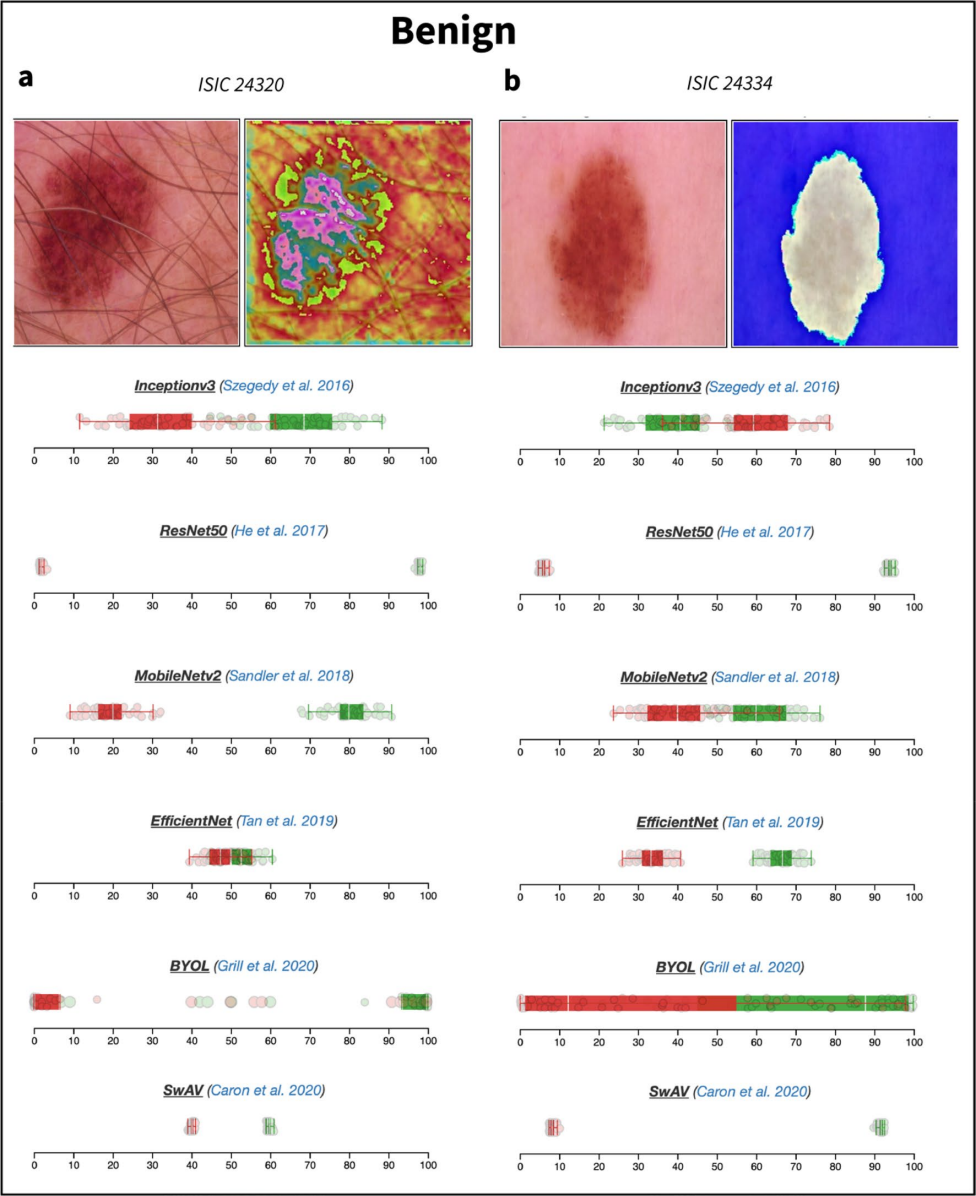
Boxplots representing uncertainty estimates provided by the six CNN models available on DUNEScan for the following skin lesion images: ISIC_0024320 (**a**) and ISIC_0024334 (**b**).

**Figure 4:**
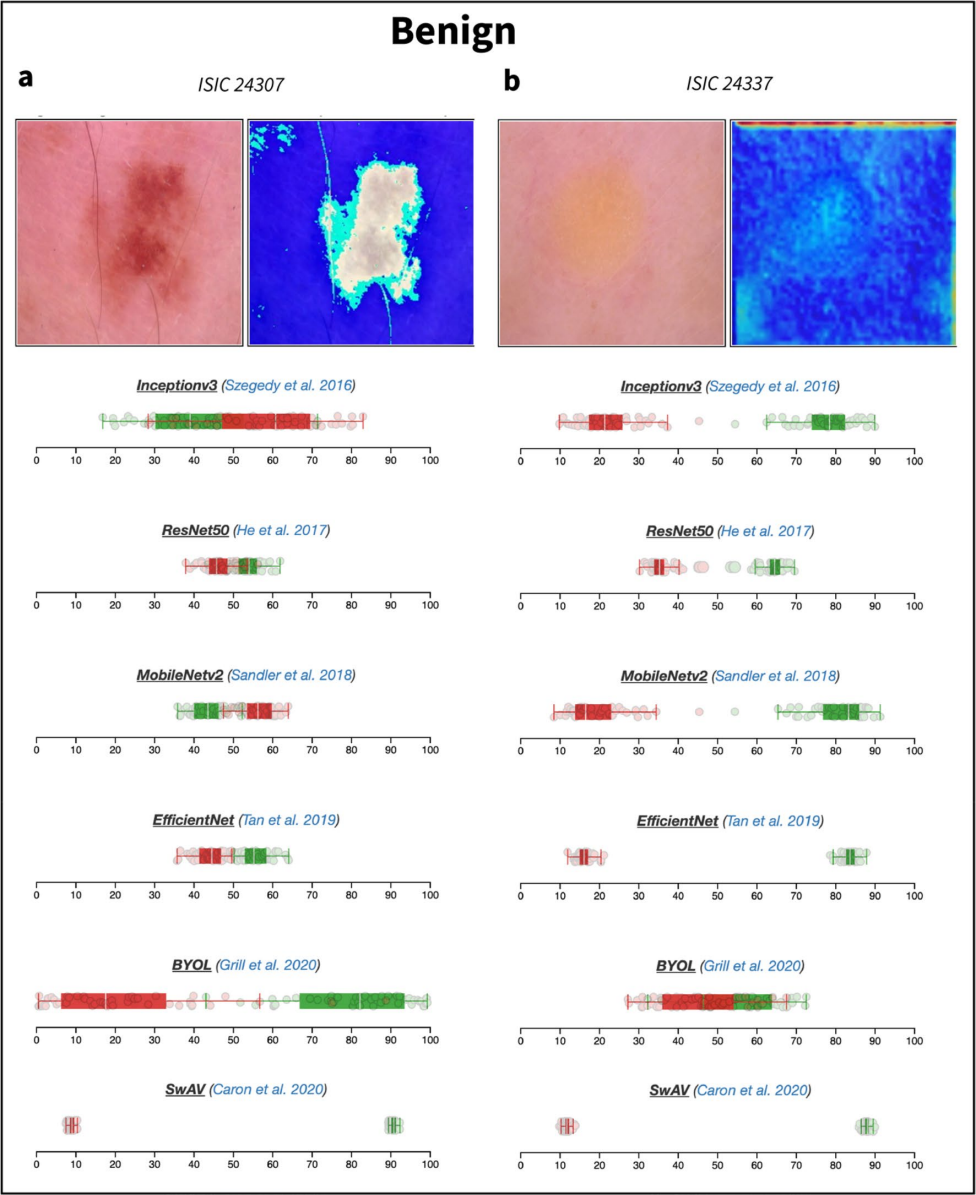
Boxplots representing uncertainty estimates provided by the six CNN models available on DUNEScan for the following skin lesion images: ISIC_0024307 (**a**) and ISIC_0024337 (**b**).

For example, for the melanoma image Mel1 (ISIC_24482) presented in Fig. 2a, all the algorithms, except BYOL, give a malignant prediction with a probability greater than 0.80 (Table 1; for improved readability, it is expressed in percentages in Figs. 1, 2, 3 and 4). As also illustrated in Figs. 2, 3 and 4, half of the algorithms (ResNet50, EfficientNet and SwAV) are highly confident in their predictions as they all output low-variance probability distributions. The MobileNetv2 and InceptionV3 models also yield reliable predictions, but the spread of their approximate posterior distribution is noticeably larger. However, the BYOL model generally provided low-confidence predictions for these images, and thus should be used with caution.

**Table 1:** Class prediction probabilities obtained for various images by the different CNN models available in *DUNEScan*.

Image name^a^	Image identifier^b^	ResNet50	EfficientNet	InceptionV3	MobileNetv2	SwAV	BYOL
Mel1	ISIC_0024482	0.95	0.81	0.81	0.81	0.96	0.01
Mel2	ISIC_0024751	1.00	0.91	0.26	0.95	0.96	0.07
Nv1	ISIC_0024320	0.00	0.48	0.27	0.12	0.36	0.03
Nv2	ISIC_0024334	0.02	0.27	0.66	0.36	0.03	0.06
Nv3	ISIC_0024307	0.45	0.43	0.53	0.58	0.03	0.1
Bkl1	ISIC_0024337	0.30	0.09	0.16	0.12	0.05	0.47

The predictions are represented as probability of malignancy, *p*(malignancy). The probability of benignancy can be obtained by 1-*p*(malignancy).

^a^Arbitrary name used as a reference in this publication.

^b^ISIC image identifier.

In the case of the melanoma image Mel2 (ISIC_24751), most algorithms yield a high probability of malignancy (above 0.90) with the exception of InceptionV3 and BYOL, which suggest that the lesion is benign with a probability of 0.74 and 0.93, respectively (see Table 1 and Fig. 2b). Although the confidence intervals produced by InceptionV3 do not overlap, they are considerably larger than those produced by the other models. Therefore, the results produced by InceptionV3 and BYOL are less reliable than the consensus prediction obtained with the rest of the models for the Mel2 image.

Interestingly, the InceptionV3 model again produces an outlier result with the melanocytic nevus image Nv2 (ISIC_24334, see Fig. 3b). In this case, all other algorithms predict that the lesion is likely benign (all producing a probability of malignancy below 0.36), whereas InceptionV3 predicts that the lesion is malignant with a probability of 0.66 (see Table 1). In this case, the two models predicting the lesion to be benign with the highest probabilities, ResNet50 (0.98) and SwAV (0.97), have the tightest prediction distribution, whereas those of both InceptionV3 and MobileNetv2 are broad and overlapping (see Fig. 3b). The distributions of the prediction probabilities obtained with EfficientNet are intermediate in size, but do not overlap. Based on these results, by relying on the models producing predictions with higher confidence (ResNet50, SwAV and EfficientNet), one could conclude that the image is indeed benign.

From the sample of melanocytic nevi images tested, it seems that the models have a difficulty producing a consensus benign prediction with high probability and confidence. Nevertheless, for most nevi images, such as Nv1 (ISIC_24320), an overall convincing set of benign prediction probabilities (all 0.52 or greater) are obtained from all models (see Table 1). The EfficientNet which produces the 0.52 probability is clearly unable to assign the lesion image to one class over the other. This is clearly illustrated by the fact that all replicate prediction probabilities for both benign and malignant classes overlap with a mean near 0.50 (see Fig. 3a). All other models, which give higher benign prediction probabilities have varying levels of confidence based on the corresponding boxplots (see Fig. 3a).

Since melanoma and nevi lesions often appear to be visually similar, this may explain why in some cases most of the models have a difficulty in favoring one class over the other. For example, with the Nv3 image (ISIC_24307, Fig. 4a) most models output predictions close to 0.50 for both classes (see Table 1). Interestingly, with this image, only SwAV classifies the lesion as benign with a high probability (0.97) and confidence (see Table 1 and Fig. 4a).

Finally, we present the results obtained with a benign keratosis (BKL) image Bkl1 (ISIC_24337, Fig. 4b), which has a clearly different appearance to those of nevi and melanoma lesions. In this case, all models except BYOL predict the lesion to be benign with a probability of 0.70 or greater (Table 1). We obtain dispersed (but non-overlapping) replicate prediction probability distributions with the InceptionV3 and MobileNetv2 models (see Fig. 4b), suggesting that the overall predictions that the lesion is benign (0.84 and 0.88, respectively, see Table 1), may not be highly accurate. However, based on Fig. 4b, the predictions from all other models, except BYOL, appear to be trustworthy. The results presented in Table 1 provide a representative sample for a handful of malignant and benign skin lesions. The confusion matrices for all six models computed for the entire test set can be found in Fig. 1.

## Discussion

Our novel web server includes six popular and well-performing CNN classification models: Inceptionv3^13^, ResNet50^14^, MobileNetv2^3^, EfficientNet^15^, BYOL^16^ and SwAV^17^. While the predictions obtained by some of these classification models disagree on a handful of outlier images (e.g., Mel2 and Nv2 in Table 1), most of them agree on prototypical images (e.g., Mel1 and Bkl1 in Table 1) of both benign and malignant skin lesion samples (i.e., lesions which lie far from the classification boundary). *DUNEScan* computes the approximate predictive posterior via dropout, which allows one to estimate this uncertainty for each model. Since dropout is applied to the latent representation, the predictive posterior’s mean can differ from the average prediction obtained without dropout. This discrepancy appears, for instance, on Fig. 1a,b, where bar plots (which represent average model prediction) do not exactly correspond to the average of the predictive posterior, even though they are very close.

An important detail which is worth mentioning is that the training set composition has a large impact on the model performance. We have constructed the training set from all labeled images of benign and malignant skin lesions available in the *International Skin Imaging Collaboration* (ISIC) archive^25^—these images originate from various datasets and hence have different camera resolutions, angles and lighting. These transformations are natural data augmentations, which have been shown to drastically improve performance on image classification benchmarks^16,26^. This partially explains a good performance of the CNN models on the test set.

While, on average, most of the six classification models have similar levels of accuracy, SwAV should be highlighted as a particularly accurate model. A striking difference in performance between BYOL and SwAV on sample images presented in Table 1 can be explained as follows: BYOL optimizes predictivity of exponentially smoothed copies of the CNN encoder, which can nevertheless collapse to the trivial representation if the smoothing parameter is too high. The advantage of the SWaV model is that it uses an equipartitioned clustering algorithm, which explicitly prevents representation collapse by requiring all clusters to have the same size. Based on this observation, we hypothesize that other clustering-based approaches can also perform well on this classification task.

One of the main limitations of our work is the size of the dataset used for training. While classical deep learning datasets tend to be fairly large (e.g. 14 million images in the ImageNet dataset^26^), medical images and skin lesion datasets, in particular, tend to be much smaller, due to their cost of collection and labelling, and medical privacy legislations. Moreover, the images contained in skin lesion datasets are usually less standardized than those in classical deep learning datasets, since these pictures are taken by different doctors, using different photographic equipment and often under drastically different lighting conditions^27^. As a consequence, conclusions which are drawn from the analysis of small datasets have to be validated by further statistical tests^9,10,28,29^ to ensure that there is clear separation between the class probabilities found for a given image.

## Conclusion

We have developed *DUNEScan*—a novel web server for assessing uncertainty of deep learning models in skin cancer detection. The main feature of *DUNEScan* is an intuitive estimation and visualization of uncertainty for the selected state-of-the-art skin cancer classifier. Uncertainty estimates are reported via boxplots of dropout replicates, Grad-CAM highlighting of “regions of interest” on the input image, as well as the projection of the input image onto the MobileNetv2 classification manifold. Thus, *DUNEScan* provides crucial information for bioinformaticians, dermatologists and health practitioners looking for an accurate skin cancer diagnosis.

## Methods

### Train-test split procedure

We created our training dataset using publicly available data from the *International Skin Imaging Collaboration* (ISIC) archive. The archive contains 23,900 skin lesion images. Among them, 2287 correspond to malignant lesions and 21,613 correspond to benign lesions. To mitigate drastic class imbalance, we combined 10,000 randomly sampled benign lesion images with all available malignant lesion images, to form a meta dataset. This meta dataset was then randomly split using a standard 80/20 train-test split from the *sklearn* Python package, under the condition that the test set had a 50–50 balance between benign and malignant cases. The validation procedure was carried out using a fivefold split of the training set. Specifically, for every training epoch, we separated the entire training set into 5 disjoint groups. Then, we trained all algorithms on 4 randomly picked groups and used the fifth one as a validation set. This validation set was used to assess the loss and the accuracy of each CNN model, in particular, to decide when to stop the training process.

Thus, our train-test split satisfies two requirements to ensure that first, the training and test sets are independent and do not have identical images, and second, that the similarity between the training and test sets is high enough so that similar patterns are included in both sets. All confusion matrices and performance metrics reported on the *DUNEScan* website were computed over this independent test set. The training procedure for both supervised and self-supervised consisted of performing a complete pass on the training set (i.e. epoch) with a mini-batch size of 16, applying stochastic data augmentation techniques such as random crops, Gaussian blurs, color jitter and rescales on every mini-batch independently. Training was performed on an NVIDIA P40 GPU with 4 CPUs, for reading the dataset in parallel. An important distinction regarding the self-supervised models (SwAV and BYOL) training procedure consisted in learning the (unsupervised) latent representation of the encoder in a first gradient step and learning the downstream binary classifier in a second step. For stability reasons, we have also used cosine learning rate annealing^30^ and Layer-wise Adaptive Rate Scaling^31^, as suggested by the SwAV and BYOL papers. Table 2 presents final average confusion metrics provided for the test set by the six CNN models available in *DUNEScan*.

**Table 2:** Average confusion metrics obtained by six CNN models included in *DUNEScan*.

	True negative (%)	False negative (%)	False positive (%)	True positive (%)
ResNet50	78.83	21.16	13.08	86.91
EfficientNet	79.27	20.72	13.67	86.32
Inceptionv3	74.29	25.7	7.47	92.52
MobileNetv2	75.31	24.68	13.51	86.48
BYOL	69.53	30.46	11.18	88.81
SwAV	70.7	29.29	11.52	88.47

### Data pre-processing procedure

For all supervised models (Inceptionv3, ResNet50, MobileNetv2 and EfficientNet), we first normalized the input image data using the traditional Z-score method with the mean value vector = [0.485, 0.456, 0.406] and the standard deviation vector = [0.229, 0.224, 0.225] taken from the *ImageNet* database (http://www.image-net.org/). We then applied the following data augmentation techniques from the *Kornia* library (https://github.com/kornia/kornia): *random flip*, *crops*, *Gaussian blurs*, *color jitter* and *rescales* for all input images in order to obtain a more balanced training set. The self-supervised models (BYOL and SwAV) were trained using the exact pre-processing described in the respective papers (see^16,17^).

### Classification manifold

The contour plot (see Fig. 1d) is obtained by extracting features of 2,000 random malignant and benign skin lesions from the ISIC databank using the MobileNetv2 network. MobileNetv2 is by far the smallest model (least memory required) among those available in *DUNEScan*, and hence its forward pass is much faster. Its output features are subsequently reduced to a 2-dimensional manifold using UMAP^32^. The hyperparameters of UMAP are those suggested by the authors of the UMAP paper^32^. They produce a clear separation between the malignant and benign classes. To remove all stochasticity from the procedure, the random seed was fixed to an arbitrary value, and the 2-dimensional point coordinates were pre-computed offline. Then, the image submitted for analysis undergoes the same normalization and feature extraction process as during training, and is finally projected on the plot as a blue dot. In our case, UMAP takes as input the pre-trained (i.e. frozen) lower-dimensional representations from the MobileNetv2 model. They are obtained by considering the before last (i.e. pre-logits) layer of MobileNetv2. We chose to use MobileNetv2 since it is the fastest of all the six available models which reduces the inference latency on the web server.

### Software used

All figures in this work were generated by the authors of this paper using the Matplotlib package^33^.
